# The threat of persistent bacteria and fungi contamination in tuberculosis sputum cultures

**DOI:** 10.4314/ahs.v21i2.18

**Published:** 2021-06

**Authors:** Grace Muzanyi, Aber peace, Bonny Wamuntu, Akol Joseph, Joanita Nassali

**Affiliations:** 1 Uganda-Case Research Collaboration; 2 Joint Clinical Research Center

**Keywords:** Bacteria, Fungi, Inoculation, PANTA (Polymyxin B, Amphotericin B, Nalidixic acid, Trimethoprim, Azlocillin)

## Abstract

**Background:**

Tuberculosis (TB) sputum culture contaminants make it difficult to obtain pure TB isolates.We aimed to study and identify persistent TB sputum culture contaminants post the standard laboratory pre-culture sample decontamination techniques.

**Methods:**

This was a longitudinal study of TB sputum culture contamination for a cohort of TB patients on standard treatment at: baseline, during TB treatment and post TB treatment. Sputum samples were decontaminated with 1.5%NaOH and neutralized using 6.8 Phosphate buffer solution.Sputum was then inoculated into MGIT (mycobactrial growth indicator tube) supplemented with 0.8ml PANTA. A drop of each positive MGIT culture was sub cultured onto blood agar and incubated for 48 hours at 35 -37OC.Any growth was identified using growth characteristics and colony morphology.

**Results:**

From October 2017 through May 2019;we collected 8645 sputum samples of which 8624(99.8%) were eligible and inoculated into MGIT where 2444(28.3%)samples were TB culture positive and 255(10.4%)were positive for contaminants: 237 none-tuberculosis bacteria, 12 fungi and 6 mixed(none-tuberculous bacteria+fungi). There was no statistically significant difference between none tuberculosis bacteria and fungi in the treatment (OR=1.4,95%CI:0.26–7.47,p=0.690) and the post treatment TB phases(OR=2.02,95%CI:0.38–10.79,p=0.411)Vs baseline.

**Conclusion:**

None-tuberculous bacteria and fungi dominate the plethora of TB sputum culture contamination and persist beyond the standard laboratory pre-culture decontamination algorithm.

## Introduction

Pulmonary TB sputum culture contamination remains a menace despite existing laboratory decontamination procedures. The contaminants may be normal oral flora, extrinsic bacteria, fungi and other none-specific organisms. The culture contaminants make it difficult to isolate TB and the process of TB culture gets more costly as it requires additional work up to isolate the TB bacilli. This results into prolonged results turnaround time, delayed diagnosis, delayed treatment and it compromises TB patient care. The mean contamination rates for specimens on liquid media at the Uganda-Case Western Reserve University Research collaboration Site in Kampala for the year 2012 was 8% for baseline samples, 12.1% for month two treatment samples,18.8%for month four treatment samples and 16.2% for month six(end of treatment) samples^2^. Past studies on interventions to reduce TB sputum culture contamination have shown significant reductions in culture contamination rates when rinsing with clean boiled water, oral antibacterial or antifungal remedies^1^,^3^.

In order to obtain pure TB cultures; the pre-culture decontamination approach should target specific TB culture contaminants at the point of sample collection and the laboratories. This would only be feasible if we are able to isolate specific contaminants so that targeted interventions can be devised to mitigate sputum culture contamination. We hypothesized that TB sputum culture contamination is caused by a mixture of organisms including bacteria and fungi that persist beyond the laboratory pre-culture decontamination techniques. We therefore aimed to study and identify the etiology of TB sputum culture contamination post the laboratory pre-culture decontamination standard techniques. This would enable laboratories and clinical teams to devise specialized decontamination interventions targeting specific organisms at the point of sample collection and the laboratory. Consquently there would be improved TB culture yield, shortened result turnaround time, faster and improved TB diagnosis, prompt initiation of TB treatment, and reliable detection of microbiological treatment outcomes.

## Methods

### Study design

This was a longitudinal study of TB sputum cultures for a cohort of confirmed TB patients at predetermined time points of: baseline, during TB treatment (weeks 2, 4, 8, 12, 17 & 26) and post TB treatment (months 9, 12, 15 & 18). The patients were adults, both HIV infected and uninfected. Patients' TB treatment lasted for 6 months and were followed up for 12 months post treatment. Sputum culture contamination data at the time points of baseline, during treatment and post treatment was compiled from October 2017-August 2019. Only mycobacterium growth indicator test (MGIT) cultures were included in this analysis. A positive TB culture was defined as a growth in MGIT that had a positive identification by mycobacterium tuberculosis (MTB) polymerase chain reaction (PCR). A contaminated culture was defined as a positive TB culture that exhibited growth on blood agar plate (BAP) sub-culture .MGIT was the TB culture media of choice given its high sensitivity in growing MTB and any associated contaminating organisms^10^. All patients rinsed with clean boiled water before sputum collection.

### Laboratory procedures

The sputum was processed using the sodium hydroxide (NAOH)/(NALC)N-acetyl-L-cysteine (Pellet supplied by Fischer scientific) method. Specimens were decontaminated with a 1.5% final concentration of NaOH for 20 minutes on a shaker set at 60 revolutions per minute (RPM) .After neutralizing the NaOH using 6.8 Phosphate buffer solution (PBS), the specimens were concentrated at 3000g in a refrigerated centrifuge for 15 minutes.The resulting supernatant was decanted leaving a small deposit of 0.5mL which was reconstituted in 1.5mL of PBS for inoculation onto culture media. About 0.5mL of the reconstituted deposit was inoculated into MGIT which had been supplemented with 0. 8ml of PANTA(Plymyxin B, Amphotericin B, Nalidixic acid, Trimethoprim, and Azlocillin.), and 0.2ml each onto 7H11S media plate (Becton, Dickinson and company) and Lowenstein Jensen tube (Becton, Dickinson and company). The PANTA used in the MGIT was reconstituted in 10mL of growth supplement.A drop of each culture that signaled positive by BACTEC MGIT 960 instrument (Becton, Dickinson and company) was subcultured onto a blood agar plate (BAP: Base powder supplied by Becton, Dickinson and company) and incubated for 48 hours at 35–37°C. The BAP were then examined for growth. Any growth recovered was identified using growth characteristics and colony morphology. TB identification was by MTB DNA PCR.

As part of the laboratory quality control/quality assurance (QA/QC) system; for all cultures, a positive control specimen (on weekly basis) and a negative control specimen (on each day of processing) were incorporated during the decontamination process along with patient samples. The positive control ensured that the decontamination process is not too harsh to the extent of killing the MTB and the media was suitable for MTB growth. The negative control checked the processing reagents contamination and monitored cross contamination of specimens.

### Ethics clearance

All the samples were collected from participants that had enrolled in TB research studies that received independent ethics review and approval. All participants had consented to have their sputum samples stored and used for any future additional studies beyond the originally stipulated research.

### Statistical analysis

The study was powered at 80% with a 5% level of precision and 5% type 1 error. It required a sample of 174 contaminated cultures but we included all the 255 contaminated cultures in the analysis to compensate for invalid samples. Data was cross tabulated, frequencies & percentages were generated. The numerically most dominant contaminant by data inspection was selected as the standard comparator. The baseline point was selected as the standard comparator of treatment and post-treatment. The logistic regression model was used to assess the variations in contaminant type across the different time points.The effect measure was the Odds ratio with its corresponding 95%CI and p-value. Of note: p-values of <0.05 and odds ratios with confidence intervals excluding 1 were taken to be statistically significant for this analysis.

## Results

From October 2017 through May 2019, a total of 8645 suspected TB sputum samples were collected of which 8624(99.8%) were inoculated into MGIT. The main reasons for excluding samples from this analysis were; no MTB growth (72%), insufficient volume (<1ml: 0.2%) or mislabeling: 0.06%).

Of the 8624 samples inoculated into MGIT, 2444(28.3%) samples were TB culture positive and all the 2444 were sub-cultured on BAP. Of the 2444 samples sub cultured, 255 (table 1) turned out to be positive for contaminants with a positive identification. Only samples from confirmed TB patients with or without HIV co-infection who had been enrolled into TB research studies, at least 3mls in volume, clearly labeled with a patient identification number(PID),collected from confirmed TB patients with the first episode of TB,consented for future research on their stored sputum samples, collected at time points of:baseline,during treatment (weeks 2,4,8,12,17,22,26) and follow up-(months 9,12,15&18) phases were eligible and included in this analysis. We found no statistically significant difference (table 2) between non- tuberculous bacteria and fungi contaminants between baseline and any of the other two phases; during treatment and post treatment/follow up. We found that irrespective of the contaminant type, contamination is lowest before TB treatment initiation, highest during the treatment phase but tappers off during the follow up phase.

**Table 1:** Variations in type of contamination by time points: N (%)

	Baseline	TB-Treatment	Post TB-treatment	Total
Bacteria	59(24.89)	105(44.3)	73(30.8)	237(100)
Fungi	2(16.67)	5(41.67)	5(41.67)	12(100)
Bacteria + Fungi	1(16.67)	3(50)	2(33.33)	6(100)
Total	62(24.31)	113(44.31)	80(31.37)	255(100)

Fisher's exact p = 0.947: Bacteria Vs fungal contaminants across time points

**Table 2:** Comparison of Bacteria contamination versus Fungi contamination across time points

	Bacteria	Fungi	OR	P-value	95%CI
Baseline	59	2	1	-	-
TB-treatment	105	5	1.40	0.690	0.26 – 7.47
Post-TB treatment	73	4	2.02	0.411	0.38 – 10.79

P-value: comparing baseline and each of the other two time points.

## Discussion

We had hypothesized that TB sputum culture contamination is caused by a mixture of organisms including bacteria and fungi that persist beyond the laboratory pre-culture decontamination techniques. Our study found that both non-tuberculous bacteria and fungi contribute to TB sputum culture contamination and persist beyond the laboratory pre-culture decontamination recipe in all the three phases; baseline, treatment and post treatment. There was no statistically significant difference in types of contaminants between non-tuberculous bacteria and fungi across all the three phases (tables 2 and 3) despite the impressive difference in absolute numbers of bacterial and fungal contaminated cultures. To our knowledge there is no study in the current published literature that has addressed the types of TB sputum culture contaminants across all the three TB phases using MGIT media and BAP post the standard lab pre-culture decontamination techniques.

**Table 3:** Comparison of Bacteria contamination versus mixed contamination across time points

	Bacteria	Mixed	OR	P-value	95%CI
Baseline	59	1	1	-	-
TB-treatment	105	3	1.69	0.654	0.17 – 16.57
Post-TB treatment	73	2	1.62	0.698	0.14 – 18.27

P-value: comparing baseline and each of the other two time points

In context of the existing literature, Kabore et al^4^ showed that oral rinse with Chlorohexidine did not reduce sputum contamination rates in Burkinafaso. In Uganda, Kalema et al^3^ showed that oral rinse with Chlorohexidine and Nystatin reduced TB sputum culture contamination rates significantly. The kabore study was on the assumption that none-tuberculous bacteria were the sole cause of TB sputum culture contamination. The Kalema study showed that fungi contributed to the TB sputum contamination plethora. Muzanye et al^1^ showed reduced rates of contamination on rinse with clean boiled water. Muzanye and Kalema^3^ studies indirectly demonstrated the presence of contaminants but our study directly identified the possible causes of TB sputum culture contamination. Rinsing with water^1^ none-selectively flashes out all possible contaminants but addition of antimicrobial rinse might reduce the contamination rates further by selectively killing/inhibiting growth of the contaminants. In many previous studies, identification of TB sputum culture contaminants was focused on bacteria without consideration of fungi as a significant contaminant^5^. For example, OMNI gene studies^6^ have always targeted bacteria as the contaminant of TB sputum cultures leaving out fungi. Most TB pre-sputum culture laboratory decontamination algorithms are designed to target non-tuberculosis bacteria as the main culture contaminant with less focus on fungi. The blood agar media used in our study targets mainly bacteria but grows about 79% of fungi^7^ implying it leaves out a significant proportion of this contaminant. The fungi contaminant detection rate could have been higher had the lab used the highly selective sabouraud agar for fungal cultures given its higher sensitivity than blood agar^8^. This is an area of further research for future studies in sub typing culture contaminants using bacterial and fungal selective media. Despite using a non selective media for fungi, our study still demonstrated that fungi are one of the key contaminants of TB sputum cultures. Our study findings are in agreement with Tushabe etal^9^ that subtyped fungal and bacterial contaminants, in TB cultures but used LJ media which is less sensitive as opposed to the highly sensitive MGIT used in our study. For solid TB cultures, a contamination rate of below 5% is desirable and for MGIT, a rate below 10%^11^ is the recommended standard. Our study had an overall contamination rate of 10.4% across all the phases despite the pre-culture decontamination techniques in the laboratory. Our findings may be a wakeup call to refocus our efforts to target both none tuberculous bacteria and fungi as equal threats to the TB sputum culture process.

The strength to our study is that it was done in a TB high burden area where there were plenty of positive TB cultures and this powered our study to detect the dominant contaminants in sputum.

## The limitations of our study

(i) It was purely a lab based study and we did not include the demographic characteristics of the TB patients who produced the sputum samples.

(ii) In addition, our study did not screen for other species of sputum culture contaminants (viruses, protozoa) or subtype the non-tuberculous bacteria or fungi like Tumushabe et al^9^ did. This is an area of further research.

## Recommendation

TB sputum culture decontamination should target at least both non tuberculous bacteria and fungi both in the laboratory and at the point of sample collection. Patients might have to rinse with clean boiled water, oral antifungal and antibacterial agents before coughing out sputum. Labaratories should add both non-tuberculous antibacterial and antifungal reagents to their TB sputum decontamination algorithms. More research is needed on further sub typing TB sputum culture contaminants with nested in phenotypic sensitivity antimicrobial testing in order to identify additional reagents required for the current TB laboratory sputum culture decontamination algorithms.

## Figures and Tables

**Figure 1:**
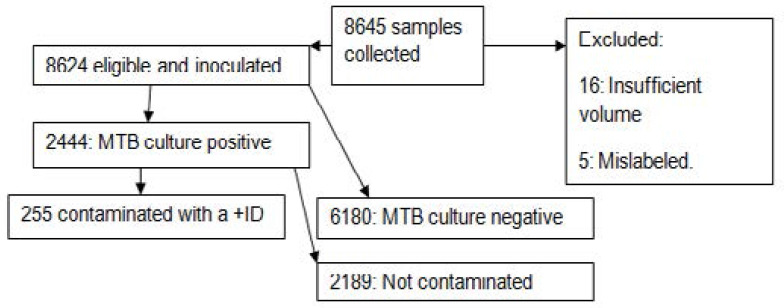
Study profile/schema
